# Constitutive androstane receptor, liver pathophysiology and chemical contaminants: current evidence and perspectives

**DOI:** 10.3389/fendo.2025.1472563

**Published:** 2025-04-04

**Authors:** Francesca De Battistis, Aleksandra Buha Djordjevic, Luciano Saso, Alberto Mantovani

**Affiliations:** ^1^ Department of Food Safety, Nutrition, and Veterinary Public Health, Italian National Institute of Health, Rome, Italy; ^2^ Department of Toxicology “Akademik Danilo Soldatović”, University of Belgrade-Faculty of Pharmacy, Belgrade, Serbia; ^3^ Department of Physiology and Pharmacology “Vittorio Erspamer”, Sapienza University, Rome, Italy; ^4^ Italian National Food Safety Committee, Rome, Italy; ^5^ Study Centre KOS - Science, Art, Society, Rome, Italy

**Keywords:** PFAS, pesticides, brominated flame retardants, liver diseases, metabolism, adverse outcome pathways

## Abstract

**Introduction:**

The Constitutive Androstane Receptor (CAR) (NR1I3), a pivotal member of the xenosensor family, plays a key role in the hepatic detoxification of xenobiotic and endobiotic chemicals through the induction of the expression of drug-metabolizing enzymes and transporters. CAR’s involvement extends beyond detoxification, influencing gluconeogenesis, lipogenesis, bile acid regulation, and cellular processes such as proliferation, tissue regeneration, and carcinogenesis. This review explores CAR regulation by various factors, highlighting its role in mediating metabolic changes induced by environmental contaminants.

**Methods:**

A literature search was conducted to identify all articles on the PubMed website in which the CAR-contaminant and CAR-hepatic steatosis relationship is analyzed in both *in vitro* and *in vivo* models.

**Results:**

Numerous contaminants, such as perfluorooctanoic acid (PFOA), Zearalenone mycotoxin, PCB, triazole fungicide propiconazole can activate hepatic nuclear receptors contributing to the development of steatosis through increased *de novo* lipogenesis, decreased fatty acid oxidation, increased hepatic lipid uptake, and decreased gluconeogenesis. Indirect CAR activation pathways, particularly involving PFOA, are discussed in the context of PPARα-independent mechanisms leading to hepatotoxicity, including hepatocellular hypertrophy and necrosis, and their implications in nonalcoholic steatohepatitis (NASH) and nonalcoholic fatty liver disease (NAFLD). The prevalence of NAFLD, a significant component of metabolic syndrome, underscores the importance of understanding CAR’s role in its pathogenesis.

**Conclusions:**

Experimental and epidemiological data suggest that endocrine disruptors, especially pesticides, play a significant role in NAFLD’s development and progression via CAR-regulated pathways. This review advocates for the inclusion of modern toxicological risk assessment tools, such as New Approach Methodologies (NAMs), Adverse Outcome Pathways (AOPs), and Integrated Approaches to Testing and Assessment (IATA), to elucidate CAR-mediated effects and enhance regulatory frameworks.

## Introduction

The “xenosensors”, or nuclear receptors for xenobiotics, pregnane X receptor (PXR) and constitutive androstane receptor (CAR) were characterized in 1998, then defined as master regulators of xenobiotic responses through transcriptional regulation of enzymes and transporters that metabolize drugs and other xenobiotics (1, 2). PXR and CAR translocate from the cytoplasm to the nucleus following ligand binding and form a heterodimer with the retinoid X receptor (RXR)α protein. The nuclear retinoid X receptor (RXR), due to its ability to form heterodimers with various other nuclear receptors such as CAR, regulates multiple cellular signaling pathways and is involved in lipid metabolism, hepatic glucose metabolism, cholesterol metabolism and bile acid homeostasis. CAR and PXR pathways have been shown to regulate CYP2/3A (Cytochrome P450 2 – 3A) expression (3, 4).

The Constitutive Androstane Receptor (CAR) (NR1I3) is among the leading members of the xenosensor family, and it is involved in the modulation of physiologic and pathophysiological conditions through the regulation of cell proliferation, energy homeostasis and tumor development. CAR can modulate gluconeogenesis, lipogenesis, and bile acid regulation, and plays a key role in the hepatic detoxification of xenobiotic and endobiotic chemicals through the induction of the expression of drug-metabolizing enzymes as well as transporters, such as bilirubin and bile acids (5–7). For instance, CAR regulates UGT1A1 (bilirubin uridine diphosphate glucuronosyl transferase, bilirubin-UGT) a key enzyme for bilirubin detoxification, which is downregulated as a result of the binding of the antagonist ligand to CAR (8). Beyond, clinically used drugs, CAR xenosensor plays a key role as a mediator of metabolic changes induced by environmental contaminants (9, 10). CAR is highly expressed in the liver and small intestine (11, 12) and it upregulates the expression of key lipogenic genes, such as fatty acid synthase (Fasn), and enzymes that limit lipid catabolism, such as carnitine palmitoyl transferase 1A (Cpt1a) (9). Activation of this receptor leads to lipid accumulation in the liver (10, 13). CAR, thus, acts at the intersection of detoxification and energy metabolism (14–16).

CAR has therefore an evident role in the physiology and pathophysiology of liver. Indeed, this multi-function organ is a main site for xenobiotic toxicokinetics/dynamics, a major actor of energy metabolism as well as an important component of the pathological phenotypes making up the metabolic syndrome. It is recognized that environmental contaminants are important risk factors for the metabolic syndrome (17), but the possible role of the interaction CAR-xenobiotics needs more attention.

The use of adverse outcome pathways (AOPs), as an innovative and established tool, is useful for elucidating associations between mechanisms and effects and the biological plausibility of epidemiological data (18–20) AOP links a molecular initiating event (MIE) to the adverse outcome (AO) via key events (KE), in a way specified by key event relationships (KER) (21). An AOP for an individual organism may include disease, developmental or reproductive alterations, whereas at the population level may include changes in population structure or local extinction of a species.

The role of xenosensors in AOP for liver diseases has raised attention by some authors (22). An AOP on the PXR activation leading to liver steatosis has recently been presented in the international AOP repository AOP-Wiki (23).

Therefore, AOP can be used to identify the possible steps to the development of liver conditions within the “metabolic syndrome” such as nonalcoholic steatohepatitis (NASH) and nonalcoholic fatty liver disease (NAFLD), as a result of CAR binding to environmental contaminants.

In this review, we explore the possible link between CAR-associated adverse effects in liver and toxicity mechanisms elicited by specific contaminants.

## Literature search

The aim of the literature search was to identify all papers on PubMed website in which the CAR-contaminant and CAR-hepatic steatosis relationship is analyzed in both *in vitro* and *in vivo* models. The literature search was performed using xenosensor, CAR, steatosis, liver as keywords and, some or all the keywords had to be present in the title or abstract of the paper. It was consulted AOP Wiki website to find out what key events had been proposed.

## Constitutive androstane receptor activation

CAR can be activated by the direct ligand-dependent mechanism or by the indirect ligand-independent mechanism that requires dephosphorylation of the receptor by protein phosphatase 2A (24, 25).

Inactivated CAR resides in the cytoplasm of hepatocytes, forming a protein complex with cytoplasmic CAR retention protein (CCRP) and heat shock protein 90 (HSP90). CAR is dephosphorylated by protein phosphatase 2A (PP2A) and translocated into the nucleus as a result of activation. Once in the nucleus, CAR regulates hepatocyte physiology through a complex network of dynamic pathways, that include;

activating the transcription of genes encoding drug metabolizing enzymes, such as UGT1A1, CYP2B6 and SULT1A1 as a result of heterodimerization with RXR and binding to the xenobiotic responsive enhancer module (26); competing with transcription factors, such as hepatic nuclear factor 4 α(HNF4α) and forkhead box protein O1 (FOXO1), for binding to the promoters of their target genes, including glucose-6-phosphatase (G6Pase) and (phosphoenolpyruvate carboxykinase 1 (PEPCK1), repressing energy homeostasis (26);

regulating cell proliferation and apoptosis through the interacting with Yes Associated Protein (YAP) -which is crucial for CAR-driven hepatocyte proliferation in the mouse (27), the stress protein growth arrest and DNA damage-inducible gene 45b (Gad45b), and beta-catenin (26).

Similar to PXR, CAR is highly expressed in liver and acts as xenobiotic receptor and regulates the transcriptional expression of many phase I and II enzymes and transporters (15, 28). Upon activation, CAR dimerizes with RXR, translocates into the nucleus, and binds to phenobarbital (PB) response element and transactivates target gene expression. While activation of PXR induces the expression of cytochrome P450 3A (CYP3As), the induction of cytochrome P450 2B (CYP2B) enzyme expression occurs as a result of CAR binding to target genes (29, 30). Coactivator proteins such as peroxisome proliferator-activated receptor gamma coactivator 1-alpha (PGC1α), growth arrest and DNA damage protein beta (GADD45β), and SRCs are involved in the transcriptional regulation of the CYP2B gene by CAR. Several studies have shown that nuclear translocation following defosphorylation of serine 202 is required for CAR activation (31); moreover, the molecular mechanism leading to nuclear translocation of CAR is mediated by peroxisome proliferator-activated receptor (PPAR)-binding protein (32). The regulation of gluconeogenesis gene expression by CAR involves several coactivators; in fact, CAR competes with FOXO1 or HNF4α for binding to insulin response elements in the promoter regions of G6pase or Pepck (33, 34). Activation of CAR inhibits liver gluconeogenesis by recruiting for ubiquitination the PGC1α, a key factor in glucose metabolism (35–38). Overall, upon activation CAR impinges in other pathways of endocrine and lipid metabolism, such as RXR, PPAR and insulin. Meanwhile, the involvement of CAR has been recognized in hepatic disorders, such as cholestatic disease (39), tumor promotion in mice (40), as well as pathways related to the metabolic syndrome (41).

Maglich showed that CAR activation results in a 50% increase in blood triglyceride levels as a result of decreased expression of PPARα target genes, a transcriptional regulator of genes associated with peroxisomal and mitochondrial β-oxidation and fatty acid transport (42). *In vivo* studies in mice have shown that CAR and PPARα compete for binding to PGC1α, a coactivator required for PPARα-mediated gene transcription (43). *In vivo* studies in healthy mice maintained on a standard diet showed that the antilipogenic action of CAR involves its activation mediated by 1,4-bis[2-(3,5-dichloropyridylory)] benzene (TCPOBOP) causing increased hepatic levels of triglycerides and cholesterol esters in WT mice but not in CAR knockout mice (13).

## CAR and fatty acid metabolism of liver: physiology and disease

One of the reasons that the mechanism for the CAR-dependent hepatocyte proliferation remains unclear is the lack of an appropriate *in vitro* system(s) to reproduce CAR-dependent cell proliferation. Unfortunately, there is no cell line reported to express CAR as strongly as in the liver or primary hepatocytes. Several studies of CAR-mediated liver tumor formation demonstrate that replicative DNA synthesis and hepatocyte proliferation are key events) although precise mechanisms on how CAR activation induces cell cycle progression of resting mature hepatocytes remain unclear. Replicative DNA synthesis and hepatocyte proliferation are also considered key events causing species differences in CAR-mediated liver tumor formation between rodents and humans. For example, phenobarbital, a well-known liver tumor promoter in rodents, activates CAR in mice, rats, and humans, but induces DNA synthesis and hepatocyte proliferation only in rodents (40).

In liver and adipose tissues, fatty acid synthesis and the subsequent generation of triglycerides takes place, a process called lipogenesis. Gene regulation of cholesterol and fatty acid biosynthesis is regulated by a group of transcription factors (SREBPs) (44–46). Lipogenesis is regulated upstream by the insulin-induced gene (Insig)-1, an endoplasmic reticulum membrane protein that, upon binding to the SREBP cleavage activator protein, results in the transport and subsequent activation of SREBP transcription factors (44, 47). CAR does impact on SREBP though its cross-talk with PPARs (48). The main PPARs cross-talking with CAR in lipogenesis is PPARα, a transcriptional regulator of genes associated with peroxisomal and mitochondrial β -oxidation and fatty acid transport (26).

Experimental models support the role of CAR in liver steatosis. The activation of CAR upregulates the expression of patatin-like phospholipase domain containing protein 3, an emerging marker of liver steatosis, as well as a panel of genes associated with glycolysis and lipogenesis, including Fasn, elongation of long-chain fatty acids family member 6 (Elovl6), stearoyl-CoA desaturase-1(Scd1), and glycerol-3-phosphate acyltransferase (Gpat) (13). This action is supported also by the patatin-like phospholipase domain containing protein 3 upregulation induced by the human CAR (hCAR) by 6-(4chlorophenyl)imidazol[2,1-b][1,3] thiazole-5-carbaldehyde O-(3,4dichlorobenzyl)oxime (CITCO) and phenobarbital (PB) in human hepatocytes *in vitro*; this action appears independent from Liver X Receptor (LXR) (13). On the other hand, Breuker et al. (49), highlighted that CAR activation by PB and CITCO and the cross-talk with PXR and RXR upregulated the lipogenic gene thyroid hormone-responsive spot 14 protein (THRSP) both *in vitro* and *in vivo*. Noticeably, THRSP expression correlates with lipogenesis and insulin sensitivity (49).

## Lifestage and sex doe influence the effects of CAR modulation

Experiments with drugs in newborn mice showed that a significant modulation of the sensitive biomarker, Cyp2 expression in liver, can persist up to adulthood upon a single treatment with phenobarbital, a CAR agonist with short half-life. On the contrary, the persistence of this effect upon treatment with another agonist, TCPOBOP, resulted from the half-life of the molecule in the liver tissue (50).

Comparative studies with Cyp -/- (Cyp3a-null and Cyp2b9/10/13-null) and CAR -/- mouse models indicated a role for CAR in the regulation of sexually dimorphic liver CYP profiles. Loss of constitutive regulation of Cyps, as a result of lack of CAR, results in changes in the expression and activity of these genes including significant repression of Cyp2a and Cyp2b members with corresponding declines in 6α- and16β-testosterone hydroxylase (51). Another study with TCPOBOP in mice showed that more than 10% of CAR-sensitive genes in liver are female-specific, genes were mainly involved in xenobiotic metabolism, inflammation, and extracellular matrix organization (52).

Meanwhile, the data obtained on CAR in rodents should be taken with caution in regard of extrapolation to humans: in mouse hepatocytes CAR induces proliferation via its interaction with YAP and is critical for liver tumorigenesis induced by PB-like compounds; on the other hand, this mechanism does not occur in human hepatocytes (53). Therefore, this CAR-mediated mechanism of action for liver tumor development in rodents is likely not relevant to humans. Even though activation of hCAR does not increase hepatocyte proliferation, rather, a reduced expression of hCAR appears to be related to a worse prognosis for hepatocellular carcinoma; in addition, the human liver carcinoma tissue shows increased DNA methylation in the hCAR promoter and lower hCAR expression, in comparison with adjacent liver tissues (26)

This gap in knowledge on the seemingly important- rodent-human differences, has motivated a shift in research toward a more comprehensive appreciation of the clinical impact of hCARs. The activation of mouse CAR inhibits hepatic gluconeogenesis and lipogenesis under nutritional challenge. In the case of human CAR, limited data so far indicate that hCAR activation also represses hepatic gluconeogenesis, while effects on lipogenesis are unclear, albeit potentially important for human health and disease (26).

*In vitro* studies on human cells of hepatocyte-origin support the important role of CAR in lipid metabolism in liver.

Overall, the available evidence supports a key role for CAR as a target of exogenous risk factors for liver disorders such as NASH and NAFLD. However, the pathways driven by CAR up- or downregulation in laboratory rodents and humans may not overlap completely.

## Environmental contaminants leading to disruption of CAR-regulated pathways

Among nuclear receptors, CAR plays a key role as a mediator of metabolic changes induced by environmental contaminants. In fact, available evidence indicate that contaminants such as PFOA, Zearalenone mycotoxin, polychlorinated biphenyl (PCB), triazole fungicide propiconazole may activate hepatic nuclear receptors contributing to the development of steatosis in terms of increased *de novo* lipogenesis, decreased fatty acid oxidation, increased hepatic lipid uptake, and decreased gluconeogenesis. The review by (54) flagged the importance of hepatic metabolism as a target for chemically-induced disruption of CAR function; meanwhile, the review pointed out that the range of toxicants affecting CAR pathways, their modes of action and the related adverse phenotypes still awaited more thorough investigations.

## Bromuconazole and other pesticides

Bromuconazole is a chiral triazole that is commonly used as a fungicide for food crops and fruits (55) and is rapidly absorbed dermally and gastrointestinally; it is then widely distributed to tissues such as liver, kidney, ovaries, and testes, and is eliminated by hepatic metabolism (56)

This fungicide can affect the PXR-CAR cross-talk in liver. Abdelhadya and colleagues (4, 26, 38, 57–59) demonstrated hepatotoxicity of the fungicide *in vivo* in rats following oral ingestion, accompanied by upregulation of PXR/CYP3A1 and downregulation of CAR/CYP2B1 gene expression. The exposure to bromuconazole resulted in hepatic oxidative damage, as evidenced by the significant decrease in superoxide dismutase (SOD) activities and significant increase in malondialdehyde (MDA) levels in the liver. The fungicide increased liver enzyme activities (ALT, AST, ALP and ACP) bilirubin levels, and liver weight, with hepatocellular vacuolization and hypertrophy. Further metabolomics and transcriptomics analysis highlighted lipid and bile acids metabolism as key targets, with an evident downregulation of PPARγ (60). Interestingly, *in vitro* experiments on human hepatocyte-derived cell lines, indicate that CAR competes with PPARα for the coactivation of PPARγ, resulting in a downregulation of PPAR-regulated pathways (43).

An interesting study investigated a mixture of six pesticides selected because frequently used for the treatment of apple orchards in the south of France: the six chemicals differed by chemical structure and mechanisms of toxicity (boscalid, captan, chlorpyrifos, thiofanate, thiacloprid, and ziram) and were present in the mixture at their respective acceptable daily intake levels. The mixture was administered via feed to WT and CAR-/- male and female mice for 52 weeks. There was a sex-specific modulation of effects. CAR-/- males did not show the increase of adiposity and the perturbation of glucose metabolism markers observed in WT males: urinary levels of pesticide metabolites and serum metabolomics were also unaffected. On the contrary, mortality was higher in CAR-/- females compared to either exposed WT mice and unexposed CAR-/- females; the levels of pesticide urinary metabolites were significantly changed, some increased and others reduced, compared to similarly exposed WT. While in WT female several PPARα-associated genes were up-regulated by pesticide exposure, a significant downregulation was observed in exposed CAR−/− females. Overall, CAR activation appeared an important mediator of adverse metabolic effects induced by the pesticide mixture, with a worsening role in males and a protective role in females. Unfortunately, the study did not provide indications on what substance(s) was mainly responsible in the CAR-mediated effects (61). Interestingly, the sex-specificity of effects was consistent with the observations by (52) on TCPOBOP-trated mice: however, in this study CAR activation enhanced liver toxicity in females.

CAR activation is also the initiating event of liver tumorigenicity in mice exposed to chlorinated pesticides toxaphene (62) and dieldrin (63): main secondary events associated with CAR activation were AhR activation (toxaphene), rising oxidative stress(dieldrin) and PXR activation (both). As already noted above, data on CAR-mediated liver tumorigenicity in the mouse should be taken with great caution, as this tumorigenic pathway is likely not relevant to humans. In the meanwhile, this evidence should not be dismissed altogether: CAR-activating chemicals might induce other, non-cancer, adverse phenotypes in human livers.

## Perfluorooctanoic acid and other perfluoroalkyl substances

Per- and polyfluoroalkyl substances (PFAS) are a class of synthetic chemicals characterized by their strong carbon-fluorine bonds, rendering them highly stable and resistant to degradation. PFAS have been used since the 1940s in various industrial and consumer applications; hence, PFAS are ubiquitous in environmental media and food chain, due to their widespread use, persistence and bioaccumulative nature. PFAS raise significant health concerns: human exposure through water, food, and air has led to measurable levels in the blood of nearly the entire population in developed countries. For instance, Perfluorooctanoic acid (PFOA) is one of the most abundant PFAS in the environment. Human exposure to PFOA occurs through drinking water, indoor dust, and food (64, 65). In addition, PFOA accumulates mainly in serum and liver (66) and has been detected in umbilical cord blood and breast milk (67–69).

Most important, PFAS are associated with a variety of adverse health effects, including altered immune and thyroid function, liver disease, lipid and insulin dysregulation, kidney disease, adverse reproductive and developmental outcomes, and cancer (70–72). The ongoing exposure levels in large groups of the European population are above the tolerable intake set by the European Food Safety Authority (73).

Interaction with PPARα is currently recognized as the main mechanism underlying PFAS toxicity (74, 75). However, several PFAS, including perfluorooctanoic acid (PFOA) and perfluorooctane sulfonate (PFOS), can also interact with CAR, leading to activation and/or modulation CAR pathways (74).

A toxicogenomic scanning using 3 mg/kg bw/day of PFOA transcript profile in mouse liver evidenced the primary involvement of PPARα. Nevertheless, PFOA additionally influenced a subset of genes independent from PPARα, particularly those related to xenobiotic metabolism, which are targeted by CAR activators such as phenobarbital and TCPOBOP. Comparing transcript profiles of PFOA-exposed mice with the profile of CAR-regulated genes revealed a strong correlation with the subset of genes involved in xenobiotic metabolism. Furthermore, exposure to PFOA in PPARα-null mice led to an increase in liver-to-body weights, indicating effects that are not reliant on PPARα activation (74). In a research investigating if perfluorocarboxylic acids (PFCAs) such as PFOA could act as CAR activators, it was found that (20 mg/kg bw/day) PFOA treatment increased mRNA levels of CAR target genes such as Cyp2b10 in wild-type but not in CAR-null mice, hence suggesting that PFOA activates CAR (76). This study also showed that PFOA treatment induced the nuclear translocation of CAR in mouse livers, a hallmark of CAR activation. The research concluded that PFOA and other PFCAs are indirect activators of CAR, functioning through a mechanism distinct from typical direct activators like phenobarbital​. Phenobarbital activates CAR by inhibiting the epidermal growth factor receptor (EGFR) signaling pathway and promoting the dephosphorylation of CAR via protein phosphatase 2A (PP2A). On the other hand, PFOA increased Cyp2b10 mRNA levels, as sensitive marker of CAR activation, even when the PP2A pathway was inhibited by okadaic acid; hence the data indicate that PFOA activates CAR through a different pathway (76)​. A bioactivity profiling of 137 nM-300 μM PFAS using transcription factor activation assays, revealed moderate efficacy of PFAS in activating the PXR and limited activation of the CAR and aromatic hydrocarbon receptor AhR (77). A recent screening of PFAS using the Tox21 approach for bioactivities on nuclear receptors, cellular stress pathways, and cytochrome p450 enzymes, revealed that inhibition of CYP2C9 was the most sensitive target (IC50 values of < 1 µM vs > 10 µM for other targets: molecular docking analysis suggested that PFAS directly bind to the active sites of CYP2C9 (26). It is noteworthy that CAR activation leads to CYP2C9 induction (78). Hence, PFOA and other PFAS might impinge on CAR-regulated genes and pathways downstream; the effect might not always be consistent with CAR activation, as downregulation can also occur. These observations imply that the action by PFOA (and other PFAS) on CAR in liver are multifaceted and the crosstalk with PPARα-dependent pathways and other nuclear receptors should be considered.

The interaction with CAR by PFAS has significant implications for the toxicological effects of these chemicals. CAR regulates various genes that play roles in drug and energy metabolism as well as in hepatocyte growth and physiology (70, 72). The interaction with CAR by PFAS will alter the expression of these genes in terms of amount and/or timing. The hepatotoxic effects of PFAS have been well-documented. PFOA, for example, has been shown to induce liver hypertrophy and increase the expression of genes associated with liver growth and detoxification. Even more important, CAR is a significant player in regulating hepatic glucose and lipid metabolism, as it modulates the expression of genes involved in glucose uptake, utilization, and fatty acid metabolism. Raised blood cholesterol level is an early changes associated with PFAS exposure in humans, although the adversity of change is unclear according to the European Food Safety Authority (75). Indeed, CAR, as well as PXR, activate Insig-1, an endoplasmic reticulum (ER) protein involved in sterol-dependent synthesis of cholesterol (79). It is thus noteworthy that the CAR-inducer TCPOBOP induces liver steatosis with accumulation of cholesterol in the hepatocytes in mice (80).

The available evidence suggests a possible role for the CAR-PFAS interaction in development of chronic liver disorders; meanwhile, recent studies highlight that the CAR-PFAS should viewed in the context of crosstalk with other receptors. The major CAR/PXR target genes, CYP2B6 and CYP3A4, were found to be significantly upregulated upon PFOA 10 and 100μM exposure in a comprehensive gene analysis of HepaRG cells. However, PFOA is unlikely to act as a direct ligand for CAR or PX; it activates the CAR/PXR signaling pathway in HepaRG cells via an indirect CAR activation pathway through its dephosphorylation (81). Indirect CAR activation pathway may be deeply involved in a PPARα-independent pathway related to the induction of hepatotoxicity, in terms of peroxisome proliferation, hepatocellular hypertrophy, necrosis. In addition, PPARα induces the expression of CAR as a negative regulator of PPARα functions in mouse liver (82); therefore, PFOA activates the expression of PPARα, which, in turn, results in the increased expression of CAR. This upregulation may also be involved in the indirect activation of the CAR pathway in HepaRG cells (81).

## Phthalates

Phthalates are a group of chemicals commonly used as plasticizers to increase plastics’ flexibility, transparency, durability, and longevity. They are found in various consumer products, including toys, food packaging, medical devices, and personal care products. Their widespread use and the fact that they are not chemically bound to the plastic and can; therefore, leak, migrate, or evaporate make them significant environmental and food pollutants and a risk to human health (73, 83, 84).

Di(2-ethylhexyl) phthalate (DEHP) is one of the most widely used phthalates as well as one of the most thoroughly investigated. It is known for its potential endocrine-disrupting effects, and humans are exposed to it through various routes, including ingestion, inhalation, and dermal contact (73, 85). DEHP exposure leads to upregulation of CAR target genes, including various cytochrome P450 enzymes such as CYP2B6 and CYP3A4 in human primary hepatocyte cultures (86). While PPARα is considered a main target of phthalates, experiments on WT and PPARα -/- mice showed that DEHP elicits a PPARα-independent regulation of a set of CAR target genes. The CAR-regulated CYPs are significantly involved in the metabolism of endogenous hormones and xenobiotics, suggesting that activation of CAR by DEHP contributes to the disruption of normal hormonal homeostasis and metabolic processes elicited by DEHP and other phthalates; among others, the prenatal exposure to DEHP elicits in post-natal mice a decreased a persistent glycogen storage in hepatocyte, accompanied by neonatal steeatosis (87). Another study highlighted the critical role of CAR and PXR in mediating the endocrine-disrupting effects of phthalates and their metabolites (88), namely the interaction between DEHP and di-isononyl phthalate (DiNP), along with their primary monophthalate metabolites, mono(2-ethylhexyl) phthalate (MEHP) and monoisononyl phthalate (MiNP), on various human CAR splice variants (hCAR1, hCAR2, and hCAR3) and PXR. The findings revealed that DEHP and di-isononyl phthalate (DiNP) are potent activators of the hCAR2 variant and, to a lesser extent, hPXR. MEHP and monoisononyl phthalate (MiNP) also exhibited significant activation of hCAR2 and moderate activation of hPXR, but only slight activation of hCAR1 and hCAR3 at higher doses. These results suggested a high specificity and potency of DEHP and its metabolites for hCAR2. Furthermore, the study demonstrated that mono-(2-ethylhexyl)phthalate (MEHP) and MiNP upregulate CAR target genes, such as CYP2B6 and CYP3A4, in primary human hepatocytes, confirming the functional consequences of receptor activation (88). The differential receptor activation by phthalates between human and rodent models underscores the importance of using human-specific data to assess phthalates’ toxicological impacts. Li et al. (89) investigated the activation of the CAR by various phthalates, including DEHP, and elucidated the species-specific differences in CAR activation. The researchers compared the effects of 15 commonly used phthalates on CAR activation using *in vitro* and *in vivo* models, including CAR knockout mice and transgenic human CAR mice. The study showed that DEHP and its metabolites are potent activators of human CAR and significantly induce the expression of CAR-regulated genes such as CYP2B6 and CYP3A4. CAR activation by phthalates leads to upregulation of enzymes such as CYP2B6 and CYP3A4, which play a critical role in metabolizing xenobiotics as well as endogenous hormones. This activation can be linked to liver-derived endocrine disruption, where excessive and/or untimely metabolism/catabolism of hormones lead to hormone deficiencies/imbalances. In addition, CAR is an important component of the orchestration of lipid, bile acid and glucose metabolism: hence, CAR activation may be important to understand and assess the possible role of phthalates in the predisposition to disorders included in the metabolic syndrome, such as obesity and chronic liver diseases: in particular, the altered lipid metabolism can exacerbate the NAFLD. The evidence assessed in the review also highlighted the species-specific differences in CAR activation, with human CAR showing higher sensitivity to phthalates than rodent CAR. Overall, it is important to understand the role of CAR in mediating the toxicological effects of phthalates; this can be relevant also for regulatory action, to understand the full spectrum of effects of human relevance and their dose-response and to identify predictive biomarkers of effects. Future studies should also investigate the combined effects of different phthalates and their potential synergistic or additive effects through CAR activation at environmentally-relevant levels, since humans are exposed to multiple phthalates simultaneously (73). While the reproductive, e.g. anti-androgenic, activities of phthalates are well-documented, less is known about their effects on other endocrine-metabolic pathways mediated through CAR. Further research is needed to elucidate the mechanisms through which phthalates disrupt thyroid and other hormone signaling pathways.

## Bisphenols

Bisphenols, a group of chemical compounds used extensively in the manufacture of polycarbonate plastics and epoxy resins, have attracted considerable attention due to their endocrine-disrupting properties. The best-known bisphenol, bisphenol A (BPA), along with its analogs such as bisphenol S (BPS) and bisphenol F (BPF), is widely used in various consumer products and leads to widespread human exposure (90–92). Research has increasingly focused on the molecular mechanisms by which bisphenols exert their effects, emphasizing their ability to interact with nuclear receptors, including CAR. Yoshihara et al. (93) investigated the metabolic activation of BPA by the S9 fraction of rat liver and its effects on estrogenic activity. While the study focused primarily on the metabolic activation of BPA and its estrogenic effects, it also provides important insights into how such processes may be related to the activation of nuclear receptors, including CAR. The results emphasized the importance of considering metabolic activation, hence the role of “xenosensors” PXR and CAR, when evaluating the endocrine-disrupting potential of BPA and other bisphenols. On the other hand. BPA does not activate the PXR *in vitro* to any significant extent (94)

Liu et al. (95) investigated the receptor binding affinities of bisphenol A (BPA) and its next-generation analogs, including BPAF (bisphenol AF), BPAP (bisphenol AP), BPB (bisphenol B), BPC (bisphenol C), BPE (bisphenol E), BPF (bisphenol F), BPM (bisphenol M), BPP (bisphenol P), BPS (bisphenol S) and BPZ (bisphenol Z) for various human nuclear receptors, 21 receptors including CAR, using competitive binding assays. The results showed that BPA and several next-generation bisphenols have a high binding affinity for CAR. In particular, BPAF and BPB were found to be highly active towards CAR with IC50 values of 5.53 nM and 6.78 nM, respectively, indicating a high potential for CAR activation. BPA also showed significant binding to CAR with an IC50 value of 10.1 nM. The strong binding of BPA, BPAF and BPB to CAR suggests that these chemicals can induce the expression of metabolizing enzymes such as CYP2B6 and CYP3A4, as well as modify pathways relevant to lipid, bile acids and glucose metabolism. The findings can be relevant to BP risk assessment as hot debate is ongoing about their implications in components of the metabolic syndrome such as NFLD and NASH (96–98). However, the possible role of a BP-CAR interaction in NFLD and NASH still awaits to be elucidated.

## Polybrominated diphenyl ethers

The Polybrominated diphenyl ethers (PBDE) are a group of 209 congeners widely in use since the early 1970s as flame retardants. PBDE are strongly lipophilic and bioaccumulate in living organisms and food chains. In addition, they are also persistent. International agreements on regulation and use of some PBDEs have been introduced since 2004. However, due to persistence and bioaccumulation, dietary exposure still poses a health concern, in Europe and elsewhere (99).

The EFSA opinion (99) summarizes evidence on the toxicology of PBDE: CAR features as a main target for PBDE hepatotoxicity. Following high levels of exposure (micromolar in cell culture), several isolated PBDE congeners (BDE-47, -99, -153, -209) as well as technical products (DE-71) can activate CAR/PXR-dependent gene expression, although PXR is more sensitive than CAR to PBDEs. Several *in vivo* (100–102) and *in vitro* (101, 103, 104) studies have shown that there is a decrease in circulating concentrations of estradiol, testosterone, and T4 as a result of CAR/PXR-dependent expression of biotransformation enzymes resulting in increased metabolism of steroid and thyroid hormones. Therefore, CAR activation in liver is one mechanism leading to indirect endocrine disruption, by increasing hormone catabolism. Liu et al. (105) have proposed to rank the potency of 49 PBDE congeners for inducing liver effects based on the affinity for the receptors most sensitive as PBDE targets, namely: AR, RXR-alpha and LXR-alpha.

**Table 1** summarizes the evidence on the environmental xenobiotics interacting with CAR, their route of exposure and adverse effects.

**Table 1:** Environmental xenobiotics interacting with CAR, routes of exposure and adverse effects.

Chemical	Route of exposure	Gene expression	Adverse Effect
Bromuconazole (Fungicide) (60)	• Dermal • Ingestion • Inhalation	• Upregulation of PXR/CYP3A1 • Downregulation of CAR/CYP2B1 gene expression	• decrease in superoxide dismutase (SOD) activities • significant increase in malondialdehyde (MDA) levels in the liver • increased liver enzyme activities (ALT, AST, ALP and ACP) bilirubin levels • increase liver weight • increase hypertrophy
Perfluorooctanoic acid (PFOA) (74)	• Dermal • Ingestion • Inhalation	• Upregulation of Cyp2b10	• increase in liver-to-body weights • induce liver hypertrophy • increase the expression of genes associated with liver growth and detoxification
Bisphenol A and Bisphenol B (90)	• Dermal • Ingestion • Inhalation	• Upregulation of CYP2B6 and CYP3A4	• alteration of blood lipid profile • increase the oxidant/antioxidant mechanism in the liver
Di(2-ethylhexyl) phthalate (DEHP) (65)	• Dermal • Ingestion • Inhalation	• Upregulation of CYP2B6 and CYP3A4	• anti-androgenic, activities • lipid accumulation in liver
Polybrominated diphenyl ethers (PBDE, BDE-47, -99, -153, -209 and technical product DE-71) (100)	• Dermal • Ingestion • Inhalation	• Upregulation of CYP3A4	• decrease in circulating concentrations of estradiol, testosterone and T4

The table summarizes the evidence of environmental xenobiotics (fungicides, perfluorooctanoic acid, bisphenols, phthalates, and polybrominated diphenyl ethers) that interact with CAR, their route of exposure, and adverse effects

## CAR-related adverse outcome pathways: present and perspectives

The use of adverse outcome pathways (AOPs), as an innovative and established tool, is useful for elucidating associations between mechanisms and effects and the biological plausibility of epidemiological data (18–20) AOP links a molecular initiating event (MIE) to the adverse outcome (AO) via key events (KE), in a way specified by key event relationships (KER) (21). An adverse outcome for an individual organism may include disease, developmental or reproductive alterations. Adverse outcomes at the population level may include changes in population structure or local extinction of a species. This approach could be useful in identifying possible factors that lead to the development of diseases such as nonalcoholic NASH and NAFLD as a result of CAR binding to environmental contaminants.

One potential application of AOP is to highlight how methods for evaluating MIE and/or early KEs can be reliably used to demonstrate that a CAR mode of action is operative for a particular molecule, avoiding the large-scale use of animal testing to demonstrate each KE in the proposed pathway. The goal is that the AOP can help the scientific and regulatory community at large recognize measurable KEs that would indicate that a xenobiotic produces hepatic effects using AOPs.

Several AOPs have already been formulated in relation to CAR activation. Specifically, AOP 107 (106) describes the activation of CAR following chronic exposure by an activating ligand (a xenobiotic as well as an endogenous compound, such as bilirubin) by analyzing the sequence of key events (KE) occurring in rats and mice that results in increased incidence of adenomas and hepatocellular carcinomas. The molecular initiating event (MIE) involves the activation of CAR; interestingly, this can occur either by direct binding (as in the case of TCPOBOP) or through an indirect ligand-independent mechanism (e.g., with phenobarbital), yet the downstream sequence of events is the same (however, lifestage-related differences are described) (50).

The subsequent translocation of CAR protein into the nucleus is accompanied by dimerization with the RXRα and binding to regulatory DNA elements of target genes. Activated CAR then alters the expression of genes (KE1) related to cell cycle control; in particular the upregulation of Ki67 and Gadd45b, genes are an indicator for CAR-driven cell proliferation in rodents, albeit the expression of these genes is not strictly CAR-dependent.

As a result, a pro-proliferative (KE2) and anti-apoptotic hepatocyte environment develops in rats and mice. This proliferative environment can give rise to increased numbers of spontaneously mutated hepatocytes, which, under continuous CAR activation, clonally expand into altered pre-neoplastic foci (KE2), forming adenomas and hepatocellular carcinomas, the adverse outcome (AO).

AOP 220 (107) describes the sequence of molecular and cellular events that induce liver cancer (AO) from prolonged activation of Cyp2E1 (MIE). The liver is the main organ devoted to xenobiotic metabolism and the series of key events affecting the production of this AO are oxidative stress (KE1), hepatocytotoxicity (KE2), and sustained/persistent cell proliferation (KE3). MIE occurs when Cyp2E1 binds a substrate, which could be CAR. Binding with CAR can trigger the subsequent cascade of events Cyp2E1 is subject to uncoupling which produces oxidative stress (KE1), and mono-oxidation of substrates produces reactive metabolites. Both reactive oxygen species and metabolites cause cytotoxicity (KE2). However, following injury, the liver is able to regenerate itself through increased cell proliferation (KE3). Under conditions of chronic activation of Cyp2E1, chronic excessive increase in levels of reactive oxygen species and cell death, and subsequent dysregulated cell proliferation, lead to tumor formation (AO).

These AOPs can be used to identify the connection between the relevant AO (hepatocellular cancer) and a CAR-related mode of action for a particular molecule, avoiding the large-scale use of animal tests to demonstrate each KE in the proposed pathway. AOP can also highlight relevant biomarkers of effect. Last but not least, the AOPs can be a valuable tool to guide future risk assessments, where dose-response values for critical early key events, e.g., NOAEL (no observed adverse effect level) or BMDL (Benchmark Dose Lower Limit), can be useful endpoints.

Unfortunately, notwithstanding the ever increasing evidence in the last decades of the major role of CAR in altered lipid homeostasis (9, 108) no AOP exists till now that recapitulates events triggered by CAR activation in relation to chronic metabolic diseases. Also, no AOP deals with adverse events following MIE different from CAR “activation, such as CAR activity modulation related to the cross-talk with other nuclear receptors, first of all PXR.

## Crosstalk between PXR and CAR

There is an established crosstalk between the two xenobiotic receptors PXR and CAR, in fact several xenobiotics eg., PBDE (EFSA CONTAM 2024), as well as drugs and steroid-like ligands are dual activators of PXR and CAR. Indeed, the model CAR activators TCPOBOP, phenobarbital and phenytoin can activate PXR as well (109). PXR and CAR share binding sites in the promoter regions of xenobiotic enzyme and transporter genes, so the functions may overlap; this redundancy could be a possible mechanism strengthening the fail-safe of xenobiotic detoxification. Both PXR and CAR are able to inhibit gluconeogenesis because they share binding to the transcription factor FoxO1, thus resulting in inhibition of binding to the insulin response element, (33).

However, the xenosensors such as CAR, PXR, PPARs regulate the expression of similar groups of genes involved in energy metabolism and bile acid homeostasis, but not in the same way; this suggests a regulatory role for several co-activators or co-repressors, which need to be further studied (110). These co-activators/co-repressors can be relevant to the fine-tuning of the specific involvement of each xenosensor in pathophysiology and toxicology. Although the physiological functions of PXR and CAR have been elucidated, the endogenous ligands, agonists or antagonists, remain to be fully defined, especially for CAR, and many of the effects caused by the activation of these receptors on hepatic pathophysiology have yet to be validated in humans.

To date, the most important challenge is to identify endogenous CAR ligands and the mechanisms involved in altering liver function in order to identify potential biomarkers and therapies in liver disease.

CAR activation causes increased expression of PPARγ and sterol regulatory element binding protein-1c (SREBP-1c) (9, 111). Expression of SREBP-1c results in up-regulation of lipogenic enzymes essential for *de novo* lipogenesis leading to increased *de novo* synthesis and lipid accumulation within hepatocytes (112–114). If the CAR agonist is not removed, lipid accumulation can result in the formation of micro- and macrovesical fat droplets within hepatocytes and cause fatty liver leading to the onset of hepatic steatosis (112–114).

Nonalcoholic fatty liver disease has become the most common cause of chronic liver disease and has an estimated prevalence of 20%-30% in the general population and 67–75% in the obese population (115–124). This disease ranges from simple steatosis to NASH and has been closely associated with obesity, insulin resistance, diabetes, metabolic syndrome, and hyperlipidemia (117, 118). The precise mechanism responsible for the development of the NASH phenotype is yet to be elucidated (Sanyal, 2005). The global prevalence of NAFLD increased by 50.4% from 25.26% in 1990-2006 to 38.00% (33.71-42.49) in 2016-2019 (p<0.001). The highest prevalence of NAFLD was in Latin America (44.37%, 30.66%-59.00%), then in the Middle East and North Africa (36.53%, 28.63%-45.22%), South Asia (33.83%, 22.91%-46.79%), Southeast Asia (33.07%, 18.99%-5.49 07%, 18.99%-51.03%), North America (31.20%, 25.86%-37.08%), East Asia (29.71%, 25.96%-33.76%), Asia Pacific 28.02% (24.69%-31.60%), Western Europe 25.10% (20.55%-30.28%) (119, 120).

A strong CAR-mediated up-regulation of the patatin-like phospholipase domain-containing protein 3 (Pnpla3) was demonstrated. Pnpla3 is a gene whose polymorphism is associated with the pathogenesis of NAFLD development. This observation was confirmed in human hepatocytes treated with the antiepileptic drug and CAR activator, phenobarbital and in immortalized human hepatocytes treated with CITCO. The molecular mechanisms controlling Pnpla3gene expression show that CAR does not act by a direct regulation of Pnpla3 transcription or via the LXR but may rather involve the transcription factor Carbohydrate Responsive Element-binding protein. These data support the biological plausibility of the observed associations between exposures to certain environmental contaminants, and lipid associated metabolic diseases (13, 125).

Metabolic derangements associated with NAFLD such as obesity and diabetes are known to have a strong underlying genetic component. Moreover, familial clustering of steatosis, NASH and cryptogenic cirrhosis has been reported, suggesting that genetic factors may contribute to the development of NAFLD (116, 126, 127). However, neither genetics nor the diffusion of “western” dietary styles can explain, alone, the rising global incidence of metabolic syndrome, including NAFLD and NASH: the involvement of environmental chemicals impinging in a number of pathways, the “metabolic disruptors”, is increasingly supported by human and experimental evidence (17). CAR definitely deserves its place among the targets of metabolic disruptors.

## Conclusions

This review is the first one, to our best knowledge, specifically addressing the role of CAR as a target of environmental xenobiotics. One main gap in order to elucidate such role, is the lack of robust mechanistic assays. Recently, Ozcagli et al. (128) proposed three *in vitro* assay test methods capable of assessing the MIE of hPPARα and hPPARγ transactivation (luciferase-based receptor transactivation assay methods using HG5LN GAL4-PPAR reporter cell lines) to altered adiposity (hMSC primary cell assay method). A similar approach could be developed for CAR ligands in the future.

From this understanding, it is possible to elucidate and support the development of appropriate and more relevant *in vitro* assay methods for humans to support regulatory applications in chemical risk assessment of adverse outcomes in obesity/metabolic disorders and to facilitate progress toward the development of testing guidelines and IATA.

The increasing knowledge show that the multi-faceted CAR functions extend well beyond the -however important- xenobiotic metabolizing pathways. Indeed, CAR is a key regulator of lipid, including cholesterol, and glucose homeostasis. The available evidence support CAR as a relevant of metabolism-disrupting contaminants that are plausibly associated with components of the “metabolic syndrome”, such as for example NAFLD and NASH.

In the meanwhile, the available evidence highlights a number of recommendations for research and risk assessment in order to respond to priority data gaps

- identify the full range of adverse health outcomes where CAR plays a pivotal role
- the role of sex and lifestage in the role of CAR in pathophysiology
- attention is focused mostly on CAR activation: is there a role for CAR downregulation
- more AOP dealing with CAR interaction in a formalized and quality-controlled way, with special attention to metabolic disruption
- identify the chemicals that recognize CAR as a highly sensitive target, and methods to screen chemicals for CAR interaction in a reliable, consistent way identify the most suitable biomarkers of effects in order to link exposure to metabolic disruptors, CAR-mediated mechanisms and surveillance of public health.

We recognize that the lack of a systematic literature search is a limitation of the present paper. Hence, we strongly recommend that a systematic literature search be conducted in order to fill the data gaps regarding the role of CAR and links to contaminants. A further limitation is the very inadequate evidence on potential target tissues other than the liver. Indeed, this limitation is highlighted in the above recommendations for further research.

The identification and assessment of metabolic disruptors is a major ongoing challenge for the whole risk assessment community (129). Strong evidence points to CAR as an important target to be considering in testing strategies and AOP development.
